# Study of Behaviors Related to Over-the-Counter Medications, in Particular Nonsteroidal Anti-Inflammatory Drugs, in the General Polish Population

**DOI:** 10.3390/healthcare14030305

**Published:** 2026-01-26

**Authors:** Kaja Kiedrowska, Agata Pawlicka, Kacper Malinoś, Emilia Sokołowska, Wojciech Marlicz, Anastasios Koulaouzidis, Norbert Czapla, Karolina Skonieczna-Żydecka

**Affiliations:** 1 Student Scientific Association of Lifestyle Medicine, Faculty of Health Sciences, Pomeranian Medical University in Szczecin, 70-204 Szczecin, Poland; kajakiedrowska@gmail.com (K.K.); 69505@student.pum.edu.pl (A.P.); 67209@student.pum.edu.pl (K.M.); 2 Student Scientific Association of Social Pharmacy, Faculty of Pharmacy, Medical Biotechnology and Laboratory Medicine, Pomeranian Medical University in Szczecin, 70-204 Szczecin, Poland; 74602@student.pum.edu.pl; 3 Department of Gastroenterology, Pomeranian Medical University in Szczecin, 70-204 Szczecin, Poland; marlicz@hotmail.com (W.M.); akoulaouzidis@hotmail.com (A.K.); 4 Department of Medicine, Svendborg Sygehus, 5700 Svendborg, Denmark; 5 Department of Plastic, Endocrine and General Surgery, Pomeranian Medical University, 72-010 Szczecin, Poland; norbert.czapla@pum.edu.pl; 6 Department of Biochemical Research, Faculty of Health Sciences, Pomeranian Medical University in Szczecin, 70-204 Szczecin, Poland

**Keywords:** adverse drug reactions, patient information leaflets, analgesic agents, nonsteroidal anti-inflammatory drugs (NSAIDs)

## Abstract

**Background**: Nonsteroidal anti-inflammatory drugs (NSAIDs) are among the most commonly used analgesics. However, their inappropriate or excessive use may lead to serious adverse effects. The aim of the study was to analyze behavioral patterns and attitudes toward the use of over-the-counter (OTC) NSAIDs, as well as the perception of risks associated with their use. **Methods**: A cross-sectional survey was conducted among 567 respondents. An anonymous questionnaire consisting of 26 items was used, addressing sociodemographic characteristics, frequency of reading drug information leaflets, frequency of NSAID use, and awareness of potential adverse effects associated with these medications. **Results**: The demographic factors significantly influenced NSAID-related behaviors. Women were significantly more likely than men to read drug information leaflets and reported more frequent use of OTC NSAIDs. Older respondents exhibited greater adherence to the principles of responsible NSAID use. Higher educational attainment was associated with more frequent and attentive reading of drug information leaflets. Urban residents reported higher median frequencies of NSAID use, whereas students demonstrated greater awareness of potential NSAID adverse effects compared with non-students. **Conclusions**: The results reveal complex patterns of NSAID consumption and underscore the need for implementing targeted public health interventions.

## 1. Introduction

Nonsteroidal anti-inflammatory drugs (NSAIDs) were first defined in the 1960s to distinguish them from glucocorticosteroids [1]. Key discoveries include ibuprofen in 1961 [2] and the mechanism of aspirin by Sir John Vane in 1971 [3,4].

Studies indicate a lack of patient awareness regarding risk factors, the consequences of alcohol consumption during NSAID therapy, potential drug–drug interactions, adverse effects, and symptoms that require discontinuation of treatment [5]. Insufficient understanding of the pharmacodynamics and safety profile of over-the-counter (OTC) medications often results in their excessive use—sometimes reaching several packages per day—which may lead to serious adverse events [6]. A major issue concerns the quality and clarity of the information provided in patient information leaflets. These documents frequently fail to address key questions regarding contraindications or drug interactions in a clear and accessible manner. Patients often experience difficulties in locating essential information, particularly when the initial sections of the leaflet do not directly address their concerns, and critical content is embedded in later parts of the text [5]. This problem may stem from the limited availability of patient-friendly leaflets, as many are primarily designed for healthcare professionals rather than for the general public [7]. Studies conducted in populations other than the general public, such as healthcare-related students, indicate different levels of awareness of NSAID adverse effects and a higher frequency of reading patient information leaflets. These findings emphasize the need to assess NSAID-related knowledge and behaviors in the general population [8,9].

This study aimed to assess NSAID use patterns, awareness of adverse effects and risks of polypharmacy, frequency of reading leaflets, and information sources among the general Polish population, to identify educational gaps.

## 2. Materials and Methods

### 2.1. Development of the Survey

The survey was conducted in the general population using an original questionnaire available in both electronic and paper formats. The questionnaire consisted of 26 items, developed to address the following behavioral aspects: (1) frequency of reading the package leaflets accompanying medications; (2) primary sources of information about medicines; (3) frequency of use of over-the-counter (OTC) nonsteroidal anti-inflammatory drugs (NSAIDs); (4) awareness of adverse effects associated with NSAID use; (5) awareness of the risks resulting from the concomitant use of two or more NSAIDs; (6) knowledge of adverse effects characteristic of NSAIDs, such as diarrhea, peptic ulcer disease, increased cardiovascular risk, acute kidney injury, and hepatotoxicity; and (7) attitudes toward potential restrictions on NSAID availability either limiting their sale exclusively to pharmacies or making them prescription-only. The questionnaire was developed based on a review of the literature and underwent pilot testing on 20 participants to assess clarity, comprehensibility, and feasibility. Feedback obtained during the pilot phase resulted in minor modifications to item wording and structure to improve clarity.

A pre-specified knowledge index (range 0–7) was constructed prior to reliability and outcome analyses using five items assessing awareness of NSAID adverse effects (diarrhea, peptic ulcer disease, cardiovascular risk, acute kidney injury, and hepatotoxicity), one item assessing awareness of the risks of simultaneous use of two or more NSAIDs, and one item on reading medication package leaflets, included as an indicator of engagement in medication safety information. Responses were dichotomized and coded. For adverse-effect items, responses indicating that NSAIDs may cause the given condition (“strongly agree” or “rather agree”) were coded as correct (1), whereas uncertainty or denial (“I do not know”, “rather disagree”, or “strongly disagree”) were coded as incorrect (0). For the item assessing concomitant NSAID use, recognition that such practice is unsafe was coded as correct. For the leaflet-reading item, regular reading was coded as correct. The 7-item knowledge index demonstrated acceptable internal consistency (KR-20 = 0.73; 95% CI 0.70–0.76), supporting the use of a composite score.

### 2.2. Participants

**Figure 1:**
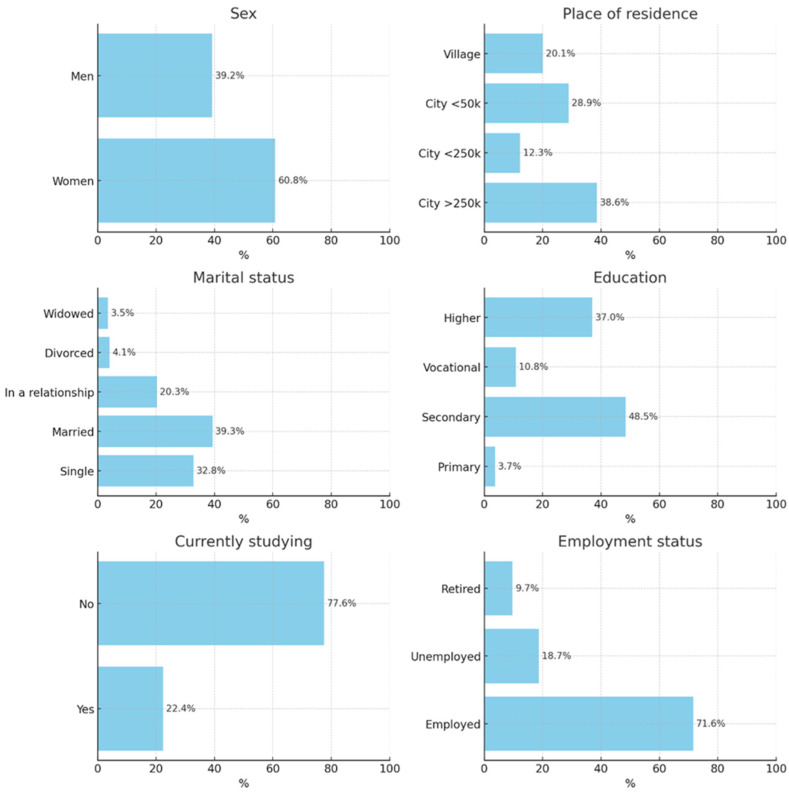
Demographic and social characteristics of the respondents.

The sociodemographic characteristics of the 567 survey respondents are presented in Figure 1. In the studied group, 22.4% of participants (*n* = 127) reported being current students, whereas 77.6% (*n* = 440) declared not pursuing any studies. Participation in the study was voluntary and anonymous. Inclusion criteria: adults aged 18+ residing in Poland. Exclusion: incomplete surveys. The sample was non-probabilistic, recruited via online and paper distribution.

### 2.3. Data Collection

Participants were recruited using non-probabilistic online and paper-based distribution. The questionnaire was distributed via the Google Forms platform (https://forms.gle/kvKCR3SCF12zB3796, accessed on 19 January 2026) and in a paper-based format. Participants were recruited using a non-probabilistic convenience sampling strategy through both online and paper-based distribution. The online questionnaire was disseminated via social media platforms and online community groups, including student- and health-related forums. Paper questionnaires were distributed in selected academic and community settings, including universities and public venues. No financial or material incentives were offered for participation. The co-authors subsequently entered the responses from paper surveys into the electronic form. Data collection was conducted between 22 November 2024 and 14 January 2025. The study did not require approval from an ethics committee, as it involved an anonymous survey and did not collect sensitive personal data. Participation was voluntary, and informed consent was obtained from all respondents at the beginning of the questionnaire.

### 2.4. Data Analysis

Statistical analyses were performed using IBM SPSS Statistics (version 30; IBM Corp., Armonk, NY, USA) to examine the relationships between demographic variables (sex, age, education level, and place of residence) and health-related behaviors. Due to overlapping response categories in the original questionnaire, NSAID use frequency was recoded into mutually exclusive ordinal categories prior to analysis. Descriptive statistics, including frequencies and percentages, were used to characterize the distribution of categorical variables. The chi-square test was applied to assess associations between categorical variables, with the level of statistical significance set at *p* < 0.05. The assumptions of the chi-square test were verified, and when appropriate, *p*-values were estimated using a Monte Carlo simulation with 10,000 replications. Effect sizes were interpreted using Cramér’s V and Phi coefficients, using standard interpretation thresholds to ensure the reliability of the results.

For group comparisons in which the assumptions of parametric tests were not met, the Kruskal–Wallis test was applied, followed by pairwise post hoc tests with Bonferroni correction to minimize the risk of Type I error resulting from multiple comparisons. From a total of 577 initially gathered questionnaires, 10 with incomplete responses were excluded prior to analysis, resulting in a final analytic sample of 567 fully completed surveys. All subsequent analyses were conducted on this complete-case dataset (*n* = 567). To illustrate the distributions of responses, group differences, and statistically significant findings, visualizations were prepared in the form of bar charts and box plots, with 95% confidence intervals applied. The minimum required sample size was calculated assuming a 95% confidence level, a 5% margin of error, and a prevalence of 50% (*n* ≈ 385); the final sample comprised 567 participants. The results were systematically presented in summary tables including test statistics, degrees of freedom, *p*-values, and effect sizes, thereby ensuring transparency and reproducibility of the study.

## 3. Results

### 3.1. Responses

A total of 577 questionnaires were collected during the study period. Of these, 10 questionnaires were excluded due to incomplete responses, resulting in a final analytic sample of 567 fully completed surveys. All subsequent analyses were conducted on this complete-case dataset. No additional exclusions were applied after data cleaning. Given the very low proportion of missing data and the consistency of the analytic sample across variables, a complete-case analysis was considered appropriate. Given the minimal level of missing data, complete-case analysis was considered appropriate, and multiple imputation was not performed. Post-stratification weighting was not applied, which may limit the generalizability of the findings.

Due to the open and voluntary nature of recruitment and the use of online dissemination channels, an exact response rate could not be calculated. For the paper-based component, questionnaires were distributed opportunistically, and the total number of individuals approached was not systematically recorded.

### 3.2. Source of Information About Medications

The results suggest that respondents prefer professional and formal sources of information about medications (physicians, package leaflets, pharmacists) over informal channels or mass media. The most frequently reported source of drug-related information was physicians, indicated by 31.2% of respondents (*n* = 177). A slightly smaller proportion of participants relied on package information leaflets enclosed with medications (29.8%, *n* = 169), while the Internet ranked third (21.7%, *n* = 123). Respondents consulted pharmacists considerably less often (10.4%, *n* = 59) or sought advice from family members or friends (5.8%, *n* = 33). Television was mentioned by only 1.1% of respondents (*n* = 6), making it the least common source of information.

The frequency of reading package information leaflets accompanying medicinal products was associated with several factors, primarily respondents’ sex (*p* = 0.011), age, and education level (*p* < 0.001). Nevertheless, the majority of participants declared that they read the contents of drug information leaflets. Specifically, 27.0% of respondents (*n* = 153) answered “definitely yes” to the question “Do you read the text of the drug information leaflet?”, while 32.1% (*n* = 182) responded “rather yes”. Another 22.8% (*n* = 129) chose “sometimes.” Negative responses were less frequent: 11.1% (*n* = 63) answered “rather no,” and 7.1% (*n* = 40)—“definitely no”.

Women were more likely than men to read medicinal product leaflets (sex differences: χ^2^(4) = 13.12, *p* = 0.011, Cramér’s V = 0.15). Female respondents significantly more often selected positive responses (“rather yes” and “definitely yes”), accounting for 59.3% and 64.7% of those indications, respectively, compared with 40.7% and 35.3% among male respondents. Although this difference was statistically significant, it should be interpreted in light of the higher proportion of women in the study sample.

A statistically significant association was found between marital status and the primary source of information about medicinal products (χ^2^ = 47.470; df = 4; *p* < 0.001). Respondents who were married most frequently indicated physicians and package information leaflets as their main sources of drug-related knowledge. This trend suggests that married individuals rely more heavily on professional and credible sources of information compared with informal channels such as family or friends. The predominance of physicians and package leaflets in this group reflects a preference for structured and evidence-based information sources (Table 1).

Respondents were divided into two age groups with respect to the frequency of reading package information leaflets: (1) <40 years and (2) ≥40 years. A statistically significant linear trend was observed (Linear-by-Linear Association = 8.739; *p* = 0.003), suggesting that the likelihood of systematically reading leaflets increases with age. The frequency of reading drug information leaflets by age group is presented in Table 2.

A strong association was also found between a higher level of education and more frequent reading of medicinal product leaflets, particularly in relation to positive behaviors such as systematically and attentively reviewing their content, as detailed in Table 3. However, no significant linear trend was observed across different education levels (*p* = 0.331).

### 3.3. Prevalence and Utilization Patterns of Non-Prescription NSAIDs

Respondents most commonly reported using over-the-counter (OTC) NSAIDs less than monthly (356/567; 62.8%), followed by monthly use (149/567; 26.3%), weekly use (58/567; 10.2%), and daily or almost daily use (4/567; 0.7%). Table 4 presents detailed data on the recoded frequency of OTC NSAID use. Women reported more frequent NSAID use than men (*p* < 0.001), particularly in the monthly, weekly, and daily or almost daily categories. Specifically, women accounted for 75.0% of the “daily or almost daily” category (3/4) compared with 25.0% men (1/4), 69.0% of the “weekly” category (40/58) compared with 31.0% men (18/58), and 78.5% of the “monthly” category (117/149) compared with 21.5% men (32/149). In contrast, the “less than monthly” category showed a near-equal sex distribution (52.0% women, 185/356 vs. 48.0% men, 171/356).

In the studied population, 24.5% of respondents (*n* = 139) reported smoking cigarettes, whereas 75.5% (*n* = 428) identified as non-smokers. After recoding NSAID use frequency into ordinal categories, a statistically significant difference was observed between smokers and non-smokers (*p* = 0.037), with smokers reporting less frequent OTC NSAID use. A significant linear trend was also identified (*p* = 0.007), supporting this pattern. This finding indicates a potential behavioral pattern but does not confirm the hypothesis of more frequent NSAID use among smokers.

**Figure 2:**
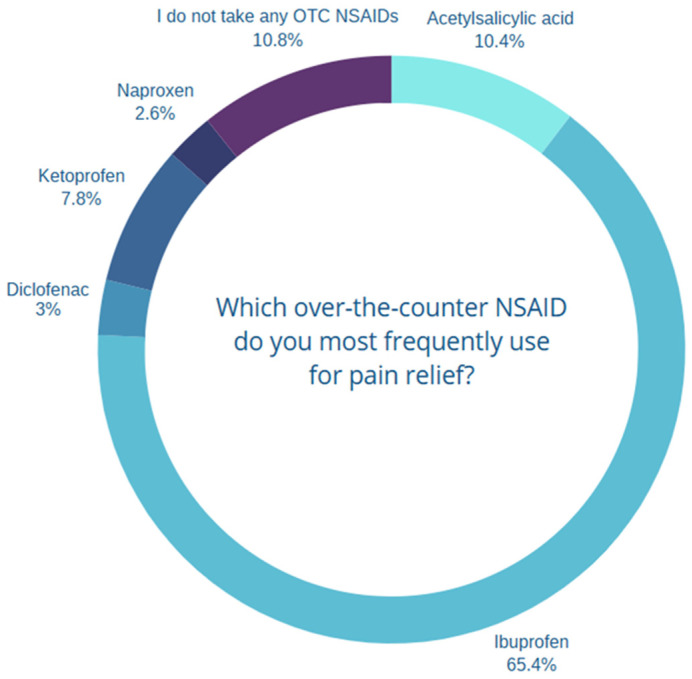
Distribution of the most frequently used over-the-counter NSAIDs for pain relief. Percentages are calculated among respondents reporting OTC NSAID use. Responses indicating the use of paracetamol or metamizole, which are not classified as nonsteroidal anti-inflammatory drugs, were excluded from the analysis. Respondents who reported not using any OTC NSAIDs are shown as a separate category.

The most commonly used OTC NSAID for pain relief was ibuprofen. Responses indicating the use of paracetamol (3.7%, *n* = 21) and metamizole (0.7%, *n* = 4), which are not classified as nonsteroidal anti-inflammatory drugs, were excluded from analyses of the ‘most commonly used OTC NSAID’ variable. The corrected distribution of OTC NSAID use is presented in Figure 2.

### 3.4. Population Awareness of NSAID-Related Adverse Effects

The majority of respondents were aware of the potential adverse effects of NSAIDs (Table 5). Statistically significant differences in responses regarding awareness of NSAID-related adverse effects were observed between students and non-students (*p* < 0.001). A detailed comparison of NSAID adverse effect awareness according to student status is presented in Table 6. Students were more likely to select positive responses, whereas non-students more frequently chose negative or neutral responses. These findings indicate differences in response patterns regarding NSAID-related adverse effects between students and non-students.

**Figure 3:**
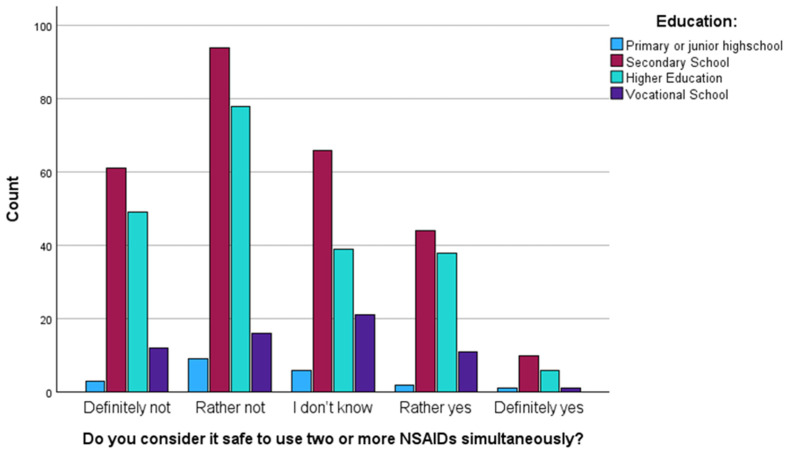
Education level and perception of the safety of using two or more NSAIDs simultaneously. Bars represent absolute counts. No statistically significant association was observed between education level and perceived safety of simultaneous NSAID use (χ^2^ test, *p* = 0.085).

The majority of respondents were aware of the risks associated with the concurrent use of two or more NSAIDs; however, one in five participants believed that such practice is safe (Table 7). No statistically significant differences were observed in the responses regarding the perceived safety of simultaneous NSAID use across different levels of education (*p* = 0.085). Nevertheless, the highest proportion of responses indicating recognition that simultaneous NSAID use is unsafe (‘definitely not’) was observed among respondents with higher education (44%) (Figure 3).

Awareness of specific adverse effects characteristic of NSAIDs is presented in Table 8.

## 4. Discussion

The aim of the present study was to analyze the relationship between selected sociodemographic variables and health-related behaviors, including the use of nonsteroidal anti-inflammatory drugs (NSAIDs) and awareness of their potential adverse effects. Previous research indicating a low level of knowledge regarding NSAID use and their widespread misuse provided the rationale for investigating this phenomenon in the general population in Poland.

The objectives of the study included (1) the development of a questionnaire and evaluation of its user testing and (2) the determination of the frequency of NSAID use, assessment of knowledge concerning their adverse effects, and analysis of the influence of sociodemographic factors on NSAID consumption in the general population.

The high prevalence of over-the-counter (OTC) NSAID use observed in the present study (89.9%) confirms that these medications are deeply embedded in everyday self-care practices in the general population. Although nearly 60% of respondents declared reading package inserts, this proportion suggests that a substantial group of users may rely on alternative information sources or personal experience rather than official safety information. Physicians were indicated as the primary source of information about OTC products by 31.2% of respondents, whereas 10.4% relied on pharmacists. Younger individuals, particularly those under the age of 40, were less likely to read the informational leaflets included with medications. The tendency to seek information about the increase in medication with age may reflect generational differences in health-related behaviors, including greater caution, accumulated experience with adverse effects, or a stronger reliance on formal medical guidance among older individuals.

Differences in NSAID-related knowledge and behaviors have also been observed when comparing the general population with more specific groups, such as students in health-related fields. Studies conducted among nursing students in Saudi Arabia reported that ibuprofen was the most frequently used NSAID, although its prevalence was lower than in the general population, reaching 20% of respondents [10]. In a study among medical university students in Palestine, 72% of the participants were aware that the concomitant use of more than one NSAID is associated with an increased risk of adverse effects [8], indicating a higher level of pharmacological awareness than that observed in the present study. Furthermore, students of medical disciplines have been shown to read patient information leaflets more frequently than students of non-medical fields (46.3% vs. 31%, respectively) [9]. These findings suggest that education in health-related disciplines may positively influence awareness of NSAID safety and information-seeking behaviors; however, such results cannot be directly extrapolated to the general population, underscoring the importance of general population-based studies.

According to a 2017 TNS OBOP survey, 73% of Poles use OTC medications, and 20% of them purchase these products outside pharmacies [11]. Koffeman et al. reported that, in the general population, 35 out of 118 individuals (30%) used OTC NSAIDs, of whom 11 (31%) reported taking two or more different NSAIDs simultaneously [12]. Arain et al. found that 68.5% of patients attending rheumatology clinics used OTC NSAIDs [13]. According to Weiner et al., 86.7% of respondents self-administered OTC analgesics. The same authors also reported that 91.1% of individuals purchased analgesics in pharmacies, although grocery stores are becoming increasingly common points of sale [14]. Data from Jalal et al. indicated that 45.4% of respondents purchased OTC medications after consulting a pharmacist, but only 3.1% reported reading the package inserts included with the products [15]. Nemata et al. found that 62.7% of participants declared reading the informational leaflets accompanying medicinal products [16].

In our study, physicians were identified as the primary source of information about NSAIDs. Similarly, in the study by Taybeh et al., 39.9% of respondents indicated physicians as their main source of information regarding OTC medications, while 34.9% cited pharmacists [17]. NSAIDs are among the most frequently misused analgesics, particularly among patients with gastrointestinal disorders. These drugs are often taken without medical supervision or prior consultation with a healthcare professional. According to Trawka et al., 7.3% of adults over the age of 60 use NSAIDs at least once a year [18]. In the study by Kozłowski et al., 12.9% of respondents reported frequent use of analgesics (once or several times per week) [19], which is comparable to our finding, where 10.2% of respondents reported a similar frequency. The most commonly used NSAID is ibuprofen [13,14,19]. In our study, 61% of respondents indicated ibuprofen as their most frequently used NSAID. The corresponding prevalence was 69.3% in the United States [13], 38.8% in Saudi Arabia [20], 65.91% in Poland according to Weiner et al. [14], 34.3% according to Kozłowski et al. [19], and only 2.04% in Egypt [21].

The most common adverse effects associated with the use of NSAIDs include nausea, vomiting, diarrhea, peptic ulcers, anemia, gastrointestinal bleeding [22], and renal dysfunction [23]. Older adults are characterized by a threefold higher risk of serious gastrointestinal events following NSAID use compared with individuals under the age of 65. However, it is important to consider the methodological limitations of cohort studies, which often result in an underestimation of the true risk of adverse drug reactions [24].

Numerous studies indicate a low level of awareness regarding the risks associated with NSAID use. Abuhamdah et al. reported that 11% of individuals incorrectly believed that OTC medications never cause adverse effects [25]. In the study by Karakitsiou et al., 45.16% of respondents were unaware of any specific adverse effects of NSAIDs [26]. Arain et al. demonstrated that 62.3% of patients were able to identify at least three adverse effects related to NSAIDs, while 18% had no knowledge in this regard. Furthermore, 79.9% of study participants were aware of the risk of NSAID-induced renal failure [13]. In the study by van den Bogert et al., 86% of respondents were aware of the gastrointestinal risks, and 67% recognized the possibility of NSAID-induced kidney disease [27]. In our study, 25% of respondents were aware of the risk of peptic ulcers, whereas only 12.9% recognized the potential for NSAID-induced renal dysfunction. This discrepancy suggests that gastrointestinal risks of NSAIDs may be more widely recognized by the public than renal complications, which are often clinically silent in the early stages. According to Tyrrell et al. NSAIDs account for 11.6% of all poisoning cases [28]. There remains a substantial gap in patient awareness of the risks associated with NSAID use—risks that are frequently underestimated and may have serious consequences, particularly among older adults with comorbidities. The nephrotoxic potential of NSAIDs was further highlighted by the “Good Aging in Skåne” study, which included 1798 participants. Among those regularly using NSAIDs, 47% had an estimated glomerular filtration rate (eGFR) below 60 mL/min/1.73 m^2^ [23], underscoring the importance of patient education regarding the adverse effects of OTC medications.

Most individuals perceive OTC medications as inexpensive and suitable for managing minor ailments [16]. The low cost of over-the-counter medical products may attract patients who prefer cheaper alternatives to more expensive formulations, regardless of their quality or efficacy. Previous studies indicate that medication cost may influence consumer decisions regarding OTC medication use [29]; however, this factor was not assessed in the present study.

Among the key strengths of this study are the large sample size and the use of a pilot-tested questionnaire available in both online and paper formats, which facilitated participation across different age groups. A limitation of the study is the sex imbalance among respondents, which may limit the generalizability of observed sex-related differences in NSAID use. Our sample may overrepresent educated individuals due to online distribution.

## 5. Conclusions

The results of the present study on the awareness and frequency of over-the-counter nonsteroidal anti-inflammatory drug use in the general population reveal significant patterns and associations.

### 5.1. Source of Information About Medications

The main sources of information about medications in the studied population are physicians and patient information leaflets enclosed with medicinal products. Individuals who are married tend to rely on these sources more frequently. The likelihood of systematically reading medication information leaflets increases with age. These findings indicate that while healthcare professionals and written materials remain important sources of patient education, their effectiveness may be limited among younger and unmarried individuals, underscoring the need for alternative educational approaches.

### 5.2. Prevalence and Utilization Patterns of Non-Prescription NSAIDs

The study highlights the association between sex, age, education level, and marital status and behaviors related to the use and perception of nonsteroidal anti-inflammatory drugs. Women use NSAIDs more frequently than men, and ibuprofen remains the most commonly used agent. While no significant associations were observed for certain attitudes, such as perceptions of the safety of concomitant NSAID use across education levels, these findings indicate that misconceptions regarding NSAID safety may be widespread and not confined to specific demographic groups. This underscores the need for broadly targeted educational interventions rather than those limited to selected subpopulations.

### 5.3. Population Awareness of NSAID-Related Adverse Effects

Most respondents demonstrate awareness of the adverse effects of NSAIDs and the risks associated with the concurrent use of two or more NSAIDs. Students exhibit greater awareness of potential adverse effects compared with non-students, suggesting a positive influence of formal education on medication-related knowledge.

The findings of this study provide a valuable foundation for developing targeted health education strategies aimed at promoting the safe and informed use of NSAIDs. Furthermore, the results emphasize the need for continued research to further examine patterns and correlates of these behaviors and beliefs, particularly in relation to demographic and sociocultural factors. These findings may support the development of targeted health education strategies aimed at younger individuals and non-student populations, including improved readability of patient information leaflets, enhanced pharmacist-led counseling at the point of sale, and public health campaigns focused on the risks of inappropriate NSAID use. Such measures may contribute to safer self-medication practices at the population level.

The use of a non-probabilistic convenience sample limits the generalizability of the findings. Post-stratification weighting to national demographic distributions was not applied; therefore, the results should be interpreted as descriptive of the study sample rather than representative of the general population.

## Figures and Tables

**Table 1:** Distribution of Primary Sources of Knowledge About Medications by Martial Status.

			What Is Your Primary Source of Knowledge About Medications?						
			Leaflet	Doctor	Pharmacist	Television	Internet	Friends or Family	Total
Martial status	Single	Count	50 _a_	52 _a_	17 _a_	2 _a_	47 _a_	18 _a_	186
		% within Martial status:	26.9%	28%	9.1%	1.1%	25.3%	9.7%	100%
	In an informal relationship	Count	35 _a,b_	24 _b_	20 _a_	0 _a,b_	28 _a,b_	8 _a,b_	115
		% within Martial status:	30.4%	20.9%	17.4%	0%	24.3%	7%	100%
	Married	Count	76 _a,b_	82 _b_	15 _a,b_	3 _a,b_	41 _a,b_	6 _a_	223
		% within Martial status:	34.1%	36.8%	6.7%	1.3%	18.4%	2.7%	100%
	Divorced	Count	5 _a_	7 _a_	5 _a_	0 _a_	6 _a_	0 _a_	23
		% within Martial status:	21.7%	30.4%	21.7%	0%	26.1%	0%	100%

Values are presented as counts and row percentages. The association between marital status and primary source of knowledge about medications was assessed using the chi-square test of independence (χ^2^(15) = 47.47, *p* < 0.001). Effect size was estimated using Cramér’s V (V = 0.17). Total sample size: *n* = 567. _a_. 0 cells (0.0%) have expected count less than 5. The minimum expected count is 15.66. _b_. Based on 10,000 sampled tables with starting seed 299,883,525.

**Table 2:** Crosstabulation of informational leaflet reading frequency by age group.

Do You Read the Informational Leaflet of a Medication?				
		Age Groups		Total
		1	2	
Definitely not	Count	28 _a_	11 _a_	39
	%Do you read the informational leaflet of a medication?	71.8%	28.2%	100%
Rather not	Count	39 _a_	24 _a_	63
	%Do you read the informational leaflet of a medication?	69.9%	38.1%	100%
Sometimes	Count	98 _a_	31 _b_	129
	%Do you read the informational leaflet of a medication?	76%	24%	100%
Rather yes	Count	104 _a_	77 _a_	181
	%Do you read the informational leaflet of a medication?	57.5%	42.5%	100%
Definitely yes	Count	83 _a_	70 _b_	153

Values are presented as counts and row percentages. The association between age group (<40 vs. ≥40 years) and frequency of reading medication information leaflets was assessed using the chi-square test of independence (χ^2^(4) = 17.79, *p* = 0.001). A significant linear trend was observed (Linear-by-Linear Association = 8.74, *p* = 0.003). Effect size was estimated using Cramér’s V (V = 0.18). Monte Carlo *p*-values were estimated using 10.000 replications. *n* = 567. _a_. 0 cells (0.0%) have expected count less than 5. The minimum expected count is 15.66. _b_. Based on 10,000 sampled tables with starting seed 299,883,525.

**Table 3:** The relationship between education level and reading medication leaflets.

Education	Definitely Not	Rather Not	Sometimes	Rather Yes	Definitely Yes
Primary or junior high school	10%	6.3%	2.3%	3.8%	2%
Secondary School	40%	41.3%	49.6%	53.8%	46.4%
Higher education	17.5%	38.1%	39.5%	35.2%	41.8%
Vocation School	32.5%	14.3%	8.5%	7.1%	9.8%
Total	100%	100%	100%	100%	100%

Values are presented as counts and row percentages. The association between education level and frequency of reading medication information leaflets was assessed using the chi-square test of independence (χ^2^(12) = 36.64, *p* < 0.001). Effect size was estimated using Cramér’s V (V = 0.15). *n* = 567.

**Table 4:** How often do you use over-the-counter non-steroidal anti-inflammatory drugs (NSAIDs)?

Frequency of OTC NSAID Use	Number of Respondents (*n*)	Percentage of Respondents
Less than monthly	356	62.8%
Monthly	149	26.3%
Weekly	58	10.2%
Daily or almost daily	4	0.7%
Total	567	100%

Due to overlapping response categories in the original questionnaire, frequency of OTC NSAID use was recoded into mutually exclusive ordinal categories prior to analysis. The recoding did not alter the underlying response data but enabled valid ordinal modeling and interpretation.

**Table 5:** Awareness of NSAID adverse effects.

Do you Believe That NSAIDs Can Cause Side Effects?	Number of Respondents (*n*)	Percentage of Respondents (%)	95% CI
Strongly yes	186	32.9%	29.1–36.8%
Rather yes	180	31.7%	28.0–35.7%
I do not know	126	22.2%	19.0–25.8%
Rather no	68	12%	9.6–14.9%
Strongly no	7	1.2%	0.6–2.5%
Total	567	100%	

Percentages are presented with 95% confidence intervals calculated using the Wilson method.

**Table 6:** Association between student status and awareness that NSAIDs can cause adverse effects (*n* = 567).

			Are You Currently Studying?		Total
			Yes	No	
Do you think NSAIDs can cause adverse effects?	Definitely not	Count	0 _a_	7 _a_	7
		% within Do you think NSAIDs can cause adverse effects?	0%	100%	100%
	Rather not	Count	9 _a_	59 _a_	68
		% within Do you think NSAIDs can cause adverse effects?	13.2%	86.8%	100%
	I don’t know	Count	14 _a_	112 _b_	126
		% within Do you think NSAIDs can cause adverse effects?	11.1%	88.9%	100%
	Rather yes	Count	45 _a_	135 _a_	180
		% within Do you think NSAIDs can cause adverse effects?	25%	75%	100%
	Definitely yes	Count	59 _a_	127 _b_	186
		% within Do you think NSAIDs can cause adverse effects?	31.7%	68.3%	100%

Values are presented as counts and row percentages. The association between awareness that NSAIDs can cause adverse effects and student status was assessed using the chi-square test of independence (χ^2^(4) = 24.54, *p* < 0.001). Effect size was estimated using Cramér’s V (V = 0.21). *n* = 567. _a_. 0 cells (0.0%) have expected count less than 5. The minimum expected count is 15.66. _b_. Based on 10,000 sampled tables with starting seed 299,883,525.

**Table 7:** Do you consider it safe to use two or more NSAID drugs at the same time?

	Number of Respondents (*n*)	Percentage of Respondents (%)	95% CI
Strongly yes	18	3.2%	1.7–4.6%
Rather yes	95	16.8%	13.7–19.9%
I do not know	132	23.3%	19.8–26.8%
Rather no	197	34.7%	30.8–38.6%
Strongly no	125	22%	18.6–25.4%
Total	567	100%	-

Confidence intervals for proportions were calculated at the 95% level.

**Table 8:** Awareness of selected NSAID-related adverse effects among respondents (*n* = 567).

	Strongly Yes	Rather Yes	I Do Not Know	Rather No	Strongly No
Do you think NSAIDs can cause diarrhea?	71 (12.5%; 10.0–15.5)	165 (29.1%; 25.5–33.0)	220 (38.8%; 34.9–42.9)	101 (17.8%; 14.8–21.2)	10 (1.8%; 1.0–3.1)
Do you think NSAIDs can cause peptic ulcer disease?	142 (25.0%; 21.6–28.8)	198 (34.9%; 31.1–38.9)	173 (30.5%; 26.8–34.5)	51 (9.0%; 6.9–11.6)	3 (0.5%; 0.1–1.6)
Do you think that NSAIDs increase the risk of cardiovascular disease in people without cardiovascular disease? (e.g., thromboembolic complications, circulatory failure)	49 (8.6%; 6.6–11.2)	151 (26.6%; 23.1–30.4)	276 (48.7%; 44.6–52.8)	88 (15.5%; 12.8–18.7)	3 (0.5%; 0.1–1.6)
Do you think NSAIDs can cause acute renal failure?	73 (12.9%; 10.4–15.9)	167 (29.5%; 25.9–33.4)	236 (41.6%; 37.6–45.8)	87 (15.3%; 12.6–18.5)	4 (0.7%; 0.3–1.8)
Do you think NSAIDs can cause liver damage?	148 (26.1%; 22.6–30.0)	248 (43.7%; 39.7–47.9)	119 (21.0%; 17.8–24.5)	51 (9.0%; 6.9–11.6)	1 (0.2%; 0.03–1.0)

Values are presented as *n* (%; 95% confidence interval). Percentages are calculated within each item. 95% confidence intervals were calculated using the Wilson score method.

## Data Availability

The raw data supporting the conclusions of this article will be made available by the authors on request.

## Abbreviations

The following abbreviations are used in this manuscript:

NSAIDs	Nonsteroidal anti-inflammatory drugs
OTC	Over-the-counter

## References

[1] Buer J.K. (2014). Origins and impact of the term ‘NSAID’. Inflammopharmacology.

[2] Halford G.M., Lordkipanidzé M., Watson S.P. (2012). 50th anniversary of the discovery of ibuprofen: An interview with Dr Stewart Adams. Platelets.

[3] Ozkan J. (2020). Sir John Vane 1927–2004. Eur. Heart J..

[4] Vane J.R. (1971). Inhibition of prostaglandin synthesis as a mechanism of action for aspirin-like drugs. Nat. New Biol..

[5] Jarernsiripornkul N., Phueanpinit P., Pongwecharak J., Krska J. (2019). Development and evaluation of user-tested Thai patient information leaflets for non-steroidal anti-inflammatory drugs: Effect on patients’ knowledge. PLoS ONE.

[6] Czekalski M., Kaczmarek P., Słomka S. (2024). Misuse of OTC drugs as a public health problem—A case report of extreme severe hypokalaemia due to OTC drug therapy. Med. Pr..

[7] Phueanpinit P., Pongwecharak J., Krska J., Jarernsiripornkul N. (2016). Medicine information leaflets for non-steroidal anti-inflammatory drugs in Thailand. Int. J. Clin. Pharm..

[8] Aboalrob A.I., Eid F.M., Esa S.M., Koni A.A., Al-Jabi S.W., Zyoud S.H. (2023). Prevalence, awareness, and patterns of non-steroidal anti-inflammatory drug use among health science students in Palestine: A cross-sectional study. Sci. Rep..

[9] Wiliński J., Lechowicz M., Kameczura T., Głowacki M., Kameczura A., Chrapusta A., Wiliński B. (2015). Non-steroidal anti-inflammatory drugs and paracetamol in self-therapy of various disorders in students of different fields of study. Folia Med. Cracov..

[10] Faqihi A.H.M.A., Sayed S.F. (2021). Self-medication practice with analgesics (NSAIDs and acetaminophen), and antibiotics among nursing undergraduates in University College Farasan Campus, Jazan University, KSA. Ann. Pharm. Fr..

[11] Woroń J. (2012). Medications Used in Self-Medication: Safety of Use and Adverse Interactions. https://www.sobieski.org.pl.

[12] Koffeman A.R., Valkhoff V.E., Celik S., W’t Jong G., Sturkenboom M.C., Bindels P.J., van der Lei J., Luijsterburg P.A., Bierma-Zeinstra S.M. (2014). High-risk use of over-the-counter non-steroidal anti-inflammatory drugs: A population-based cross-sectional study. Br. J. Gen. Pract..

[13] Arain A., Rasheed M., Sallam N., Sarwar Z., Khan M. (2019). Patient’s knowledge and use of oral non-steroidal anti-inflammatory drugs in a rheumatology clinic. Kans. J. Med..

[14] Weiner M., Tokarska-Rodak M., Bida A. (2019). Use of Analgesic Medications Across Age Groups with Consideration of Intergenerational Differences. Rozpr. Społeczne.

[15] Jalal S.M., Jalal S.H. (2024). Public Awareness and Practice Regarding Over-the-Counter Medications: A Cross-Sectional Study in Al-Ahsa, Saudi Arabia. Cureus.

[16] Nemat A., Rezayee K.J., Essar M.Y., Mowlabaccus W.B., Ahmad S., Mubarak M.Y. (2023). A report of Kabul internet users on self-medication with over-the-counter medicines. Sci. Rep..

[17] Taybeh E., Al-Alami Z., Alsous M., Rizik M., Alkhateeb Z. (2019). The awareness of the Jordanian population about OTC medications: A cross-sectional study. Pharmacol. Res. Perspect..

[18] Vandraas K.F., Spigset O., Mahic M., Slordal L. (2010). Non-steroidal antiinflammatory drugs: Use and co-treatment with potentially interacting medications in the elderly. Eur. J. Clin. Pharmacol..

[19] Kozłowski P., Cuch B., Kozłowska M., Kozłowsk K., Jędrzejewska B. (2015). Analysis of habits and behaviours related to the use of over-the-counter painkillers. J. Educ. Health Sport.

[20] Almohammed B.A. (2023). Frequency and Knowledge of Analgesics Self-Use and Their Adverse Effects in the Eastern Province of Saudi Arabia. Cureus.

[21] Kamal M., Negm W.A., Abdelkader A.M., Alshehri A.A., El-Saber Batiha G., Osama H. (2023). Most common over-the-counter medications and effects on patients. Eur. Rev. Med. Pharmacol. Sci..

[22] Sostres C., Gargallo C.J., Lanas A. (2013). Nonsteroidal anti-inflammatory drugs and upper and lower gastrointestinal mucosal damage. Arthritis Res. Ther..

[23] Modig S., Elmståhl S. (2018). Kidney function and use of nonsteroidal antiinflammatory drugs among elderly people: A cross-sectional study on potential hazards for at risk population. Int. J. Clin. Pharm..

[24] Gabriel S.E., Jaakkimainen L., Bombardier C. (1991). Risk for serious gastrointestinal complications related to use of nonsteroidal anti-inflammatory drugs: A meta-analysis. Ann. Intern. Med..

[25] Abuhamdah S.M.A., Naser A.Y. (2024). Self-medication practice among the general public in Jordan: A cross-sectional study. Front. Public Health.

[26] Karakitsiou M., Varga Z., Kriska M., Kristova V. (2017). Risk perception of NSAIDs in hospitalized patients in Greece. Bratisl. Lek. Listy.

[27] van den Bogert C.A., Miller M.J., Cobaugh D.J., Chen L., Allison J.J., Saag K.G. (2017). Screening Questions for Nonsteroidal Anti-inflammatory Drug Risk Knowledge. J. Patient Saf..

[28] Tyrrell E.G., Kendrick D., Sayal K., Orton E. (2018). Poisoning substances taken by young people: A population-based cohort study. Br. J. Gen. Pract..

[29] Aufegger L., Yanar C., Darzi A., Bicknell C. (2021). The risk-value trade-off: Price and brand information impact consumers’ intentions to purchase OTC drugs. J. Pharm. Policy Pract..

